# Predictors and motives for mask-wearing behavior and vaccination intention

**DOI:** 10.1038/s41598-023-37072-6

**Published:** 2023-06-25

**Authors:** Jakub Binter, Ondra Pešout, Michał Pieniak, Judit Martínez-Molina, Edward J. Noon, Michal M. Stefanczyk, Stephanie J. Eder

**Affiliations:** 1 Faculty of Social and Economic Studies, Jan Evangelista Purkyně University, Moskevská 54, 400 96 Ústí nad Labem, Czech Republic; 2 Department of Philosophy and History of Science, Faculty of Science, Charles University, Prague, Czech Republic; 3 Department of Psychology, Jan Evangelista Purkyně University, Ústí nad Labem, Czech Republic; 4 Institute of Psychology, University of Wroclaw, Wroclaw, Poland; 5 Faculty of Psychology, University of Barcelona, Barcelona, Spain; 6 Institute of Childhood and Education, Leeds Trinity University, Leeds, UK; 7 Department of Neurosciences and Developmental Biology, University of Vienna, Djerrassiplatz 1, 1030 Vienna, Austria

**Keywords:** Human behaviour, Infectious diseases, Lifestyle modification

## Abstract

Containing a pandemic requires that individuals adhere to measures such as wearing face-masks and getting vaccinated. Therefore, identifying predictors and motives for both behaviors is of importance. Here, we study the decisions made by a cross-national sample in randomized hypothetical scenarios during the COVID-19 pandemic. Our results show that mask-wearing was predicted by empathic tendencies, germ aversion, and higher age, whilst belief in misinformation and presentation of an interaction partner as a family member lowered the safety standards. The main motives associated with taking the mask off included: rationalization, facilitating interaction, and comfort. Vaccination intention was positively predicted by empathy, and negatively predicted by belief in misinformation and higher costs of the vaccine. We found no effect of immunization status of the surrounding social group. The most common motive for vaccination was protection of oneself and others, whereas undecided and anti-vaccine groups reported doubts about the effectiveness and fear of side effects. Together, we identify social and psychological predictors and motives of mask-wearing behavior and vaccination intention*.* The results highlight the importance of social context for mask-wearing, easy access to vaccines, empathy, and trust in publicly distributed information.

## Introduction

### The COVID-19 pandemic in Europe

The world-wide Coronavirus Disease 2019 (COVID-19) pandemic continues to threaten the physical well-being of people around the world^1^. Whilst the first pandemic wave initially hit Europe in the spring of 2020, the pandemic trajectories differed between individual European countries. For example, Spain had extremely high infection rates and mortality in the first phase, where dramatic pictures of public places used as morgues haunted the nation^2^. Similarly, the United Kingdom experienced severe strains on the medical system^3^. On the other hand, countries such as Austria reported only moderate case numbers^4^, and some countries, such as the Czech Republic and Poland, at first managed to contain the pandemic with very low case numbers^4^. It is likely that these markedly differing experiences during 2020 left a lasting impression on citizens in terms of fear of the virus and general insecurity with regard to the pandemic.

### Individual behavior can shape the pandemic

To help contain the pandemic, measures such as social distancing, wearing of face-masks, and even curfews were enforced throughout Europe^4–8^. Wearing face-masks avoids the spread of aerosols (i.e., small droplets carrying the virus^9^). However, an important aspect of the cloth mask most commonly worn at the beginning of the pandemic is that the mask does not protect the wearer directly, but rather, the created barrier mostly shields interacting partners^10^. In line with this, persons exhibiting more empathic traits tend to be more willing to wear face-masks^11^, and pro-social orientation is one of the strongest predictors of mask wearing^12^. On the other hand, masks can cause physical and psychological discomfort to the wearer, where overheating and breathing obstruction are frequently reported^13^. In addition, some perceive a loss of individuality in appearance, or perceive obeying to mask mandates, as a threat to their autonomy. The latter aspect differs between sexes and is more common in men^14,15^.

More generally, individuals high in perceived vulnerability to disease are more likely to comply with the rules that might protect them^16–18^.

Workplaces and interaction with family members is the main source of infection with the virus causing COVID-19^19^. Importantly, there are different measures of safety at workplaces compared to homes, differing mechanisms of control, and differing expectations from interaction partners. For example, interaction with a relative as compared to a stranger is of a different nature, with more trust being exhibited towards the relative^20^. Nevertheless, during most interactions, signaling trust (that the other is a safe partner for interaction) and creating a comfortable atmosphere are elementary to sustain affiliative relationships and familiar bonds^21,22^. The COVID-19 pandemic has made these interactions more difficult: Often, there are uncertainties about the infection risk coming from a person, and personal relationships have been strained by different attitudes toward safety measures against the virus.

### Not throwing away my shot: Vaccination as a pro-social act

A scientific advance with the potential to end the COVID-19 pandemic are vaccines^23^. Vaccines protect not only the vaccinated party from a severe course of the disease, but a vaccinated individual is also a safer interaction partner for others^24^*.* Importantly, a high proportion of vaccinated individuals in a social group also lower the threat of contamination for ‘at-risk’ persons (whose immune system might not be able to build up effective immunization in response to the vaccine)^25,26^. Thus, vaccinating a high proportion of a population can slow down the viral spread and gradually build herd immunity within the population^27^. However, despite large campaigns to communicate the benefits of vaccines^28^ and historically unmatched media coverage^29^, many misconceptions surround this topic. Possibly, the dynamics of both viral spreading and immunization on a group level appear counter-intuitive, since medical interventions traditionally rely on single-individual treatment and well-being^27^.

Worldwide campaigns on vaccination and their potential societal hurdles have been discussed long before the COVID-19 pandemic^30^. Currently, multiple correlates of vaccination tendency have been identified. Sociodemographic variables such as higher age^31,32^, fear of the disease/perceived vulnerability (for COVID-19^33^), and belief in the safety of the vaccine are reported as correlates of high vaccination intentions, alongside the intention of protecting others by being vaccinated^34,35^. Political and social factors such as a sense of community are debated: Possibly, a higher sense of community in the population relates to less ‘free-riding’ (i.e., profiting from herd immunity without contributing to it) and higher vaccination rates^36,37^. Individuals who focus their attention on the dangers, rather than benefits, of vaccines also tend to avoid other activities oriented at collective benefit such as mask wearing and ‘social distancing^38,39^. Overall, higher identification with one’s social group (community/nation) is beneficial for rule compliance and diminishes the impact of misinformation^40,41^. Moreover, trust in official institutions elevates vaccination intentions^42^, where media play an important role regarding the relationship between institutions, scientific findings, and citizens^42^. Unfortunately, individuals who are worried about the dangers of the novel virus often obtain their information via unevaluated sources and fall victim to *fake news* information, leading to disbelief in the benefits of vaccination or fear of possible side-effects^43,44^.

Taken together, protective measures to defeat the ongoing pandemic (such as vaccination) are effective mostly at a group level, and, in the case of regular mask-wearing, are mostly beneficial to other individuals rather than to the actor. Thus, individuals failing to comply with such measures can be seen as ‘free-riders’ in the population (e.g.,^38,45^). Identifying predictors and motives of adherence to pro-social safety regulations enables the design of evidence-based interventions, and is possibly the most important step towards avoiding free-riders and putting an early end to pandemic states.

### Free-riding in game theory

Game theory models situations where multiple players compete in a joint social setting, and has been applied to areas as diverse as energy consumption, ecology, and moral choice^46,47^. Importantly, each player is driven by self-interest, i.e., trying to minimize costs and risks, while maximizing pay-off^48^. However, the globally best outcome can differ from individual optima, and often cooperation is the key to public success, but not necessarily individual success. A prominent example are public goods games, where the globally best outcome relies on contributions from (costs to) all players of a group. As soon as enough players contribute, the scenario allows for free-riding, the possibility for an individual to not bear any costs while enjoying the full pay-off. However, if the amount of free-riders maximizing short-term self-interest becomes too high, the system collapses and the pay-off is low or nil for everyone.

Detrimental outcomes of non-cooperation also emerge in two-player scenarios. Famously, in the prisoner’s dilemma, two players need to trust each other to both make a cooperative move. The individual highest pay-off relies on the partner cooperating, while not cooperating oneself, whereas the globally best outcome results from cooperation on both sides. Cooperation bears the risk of a non-cooperative partner (resulting in the individually worst outcome).

However, human decision-making does not follow mathematically optimal solutions^49,50^, and behavioral outcomes are influenced by both situation- and person- specific factors. Thus, we need to consider the interplay between situation, inter-individual variance in personality, and social context (e.g.,^51^) when finding predictors of behaviors that can be described as free-riding, altruistic contributions, or a mutually beneficial cooperation in the context of avoiding infectious diseases. In this research, we focused on identifying predictors of and motives for concrete health-beneficial behaviors depending on social context.

### The current study

The present study aims at identifying predictors of vaccination intentions and mask wearing behavior in five European countries, each of which differed with regards to how much the pandemic affected the population in the year prior to the study: Czech Republic (CR), Poland (PL), United Kingdom (UK), Spain (ES), and Austria (AT). We employed hypothetical scenarios of varying social context presented via online-questionnaires, where participants had to make decisions regarding mask-wearing and vaccinations. Our design was inspired by evolutionary game theory (e.g.^45^), where each scenario offered three alternative behaviors: free-riding, cooperative, and altruistic actions.

We hypothesized that trustworthiness of the interaction partner and empathy plays a role in the decision to wear face-masks in different social situations. We therefore studied how prior information about a hypothetical interaction partner, relationship with the partner, partner’s actions, and psychometric parameters of the participant influence decisions in scenarios about mask-wearing. Second, we aimed to study which social and psychometric variables influence vaccination intention in a hypothetical scenario, where an individual can either actively get vaccinated while also bearing costs of a vaccine (monetary and side effects) or passively be protected by herd immunity. Possibly, immunization status of the surrounding group influences this decision: ‘free-riding’ is only possible if the group is already immunized, however, social pressure is conversely known to be a major factor in health-related decision making^52,53^.

Our hypotheses state that:i) Regarding mask-wearing, respondents will report riskier behavior when interacting with a family member as compared to a stranger,ii) Prior information about the interacting partner related to infection risk will influence mask-wearing behavior (higher risk leading to less tolerance for the partner taking their mask off, and a lowered probability to take the own mask off),iii) Vaccination intentions will be higher if the costs (both monetary and side effects) are lower, andiv) Decisions in both scenarios will be directionally modulated by the following variables:

- Empathy with vulnerable groups (positively predicting mask-wearing and vaccination intentions)
- Perceived vulnerability to disease (consisting of the subscales: infectability and germ aversion, both positively predicting mask-wearing and vaccination intentions), and
- Belief in misinformation about both the virus and protective measures (predicting lower mask-wearing and vaccination intention).

Our explorative questions include: i) How will immunization status of the surrounding group affect vaccination intention?, ii) Which decision motives (reasons) will be reported by groups with low/high vaccination intention and mask-wearing behavior?, iii) Will the participants’ sex predict compliance and cooperation in these scenarios?, and iv) How will national background affect decision-making?

In summary, our quantitative and qualitative analysis provides novel insights into the motives underlying safety-related decision making.

## Methods

### Participants

Our final sample consisted of 296 respondents (min. age = 18, mean age = 35.27, *SD* = 13.28; *n* = 183 female). The participants came from five European countries: Czech Republic (*n* = 56; 33 female), Poland (*n* = 77; 48 female), United Kingdom (*n* = 61; 37 female), Spain (*n* = 52; 31 female), and Austria (*n* = 50; 34 female). Importantly, the samples are comparable based on age (χ2(4) = 7.230, *p *= 0.13) and level of education (χ2(4) = 9.124, *p *= 0.58). For detailed information, see Table S1. Cases with an item-non-response rate greater than 5% have been excluded from this final sample (n = 4)^54^. Further, we employed three attention checks within the questionnaire (e.g., “please mark the highest point on this scale”) and excluded 36 participants who failed to provide the right answers. Moreover, data from participants residing in a country not investigated in this study have been excluded from analyses (n = 25).

### Vaccine rollout and vaccination rate against COVID-19 in the sample

Data were collected in April 2021, during the early phase of vaccine rollout which mainly aimed at first protecting vulnerable groups, and eventually containing the COVID-19 pandemic across the studied countries (see Fig. S1;^55^). 31.1% of participants were already vaccinated, whilst 51% of participants expressed their desire to do so as soon as they were able to. However, 2% were vaccinated due to their profession, but would have avoided it if possible, and 16% reported that they would avoid the vaccination at all costs. Of all participants, 69% believed that the available vaccines have many side effects, but only 28% of all participants believed that the side effects outweigh the benefits.

### Mask mandates in the studied countries

At the time of data collection, mask wearing was required only in specific public places in three of the studied countries (AT, ES, PL), and recommended in the two other countries (UK, CR). However, all of the countries had faced more stringent mask mandates in the preceding months. It is noteworthy that worldwide consensus both in populations and governments about the efficiency of mask wearing varied over time, however, it was the recommended policy in all of the sampled countries at the time of the study.(see Fig. S2;^55^).

### Procedure

Participants were recruited mostly via social media (convenience sample) and filled in an online questionnaire hosted by the SoSci platform (www.sosci.de), which included imaginary scenarios that called for action choices. After providing online informed consent, participants were presented with two consecutive “mask scenarios” twice. The first two scenarios employed a different hypothetical interaction partner than the second two scenarios, leading to four mask-related decisions being made. Participants also answered questions about vaccination intentions and socio-demographic information; and filled in questionnaires regarding traits that potentially modulated how likely they were to wear masks and get vaccinated. The data were collected during a two-week period at the end of April 2021 to ensure comparability within countries.

### Mask scenarios

Participants were asked to imagine scenarios where a) status of the interacting partner (family member/stranger) and b) prior information about the interacting partner (trustworthy and low risk *or* high infection risk *or* no information) were manipulated. They first received the following prompt: “Please imagine that you are asked to meet a cousin/potential coworker” (variable a), followed by one of three prompts about the interacting partner (variable b). Each participant was presented with two consecutive scenarios where they were interacting with a family member, and additionally two consecutive scenarios where they were interacting with a potential co-worker. The order was randomized. In both scenarios, prior information about the interacting partner was randomized (see Table S2).

Participants were then asked: “You will meet for about an hour indoors. How would you decide in the following situations?”.

On the following page, participants received the following prompt for the first mask scenario: “After you enter the room, you take your jacket off and seat yourself by a small coffee table. Do you…”. Participants could now choose whether to i) keep their mask on (“to ensure safety and compliance with the regulations”), representing the safe and cooperative action, or ii) take it off (“to ensure comfort and ease of interaction”), a unilateral decision that puts the interacting partner at risk.

After participants answered this question, they were confronted with the following second mask scenario: “Imagine that, in the same scenario, the other person decides to take the mask off their face to allow for comfort and ease of interaction with you. Do you…”. Here, participants were presented with four options, where comfort and risk varied for both partners: A) to keep their mask on regardless and be put at risk unilaterally while gaining no comfort, B) decide to take their own mask off, too, resulting in equal risk and comfort for both partners, C) insist that the other person puts the mask back on, ensuring equal safety, and D) suggest that the other person puts the mask back on but take their own mask off, which is the opposite of A). As opposed to the first mask scenario, here, the safest option (C) requires action of the participant against the fictional interacting partner. Thus, this scenario promotes an active behavior from the participant. The partner’s motives in the scenario (“comfort”) suggests an egoistic move on the partner’s side while the participant bears all costs, which however might also emotionally bias participants against the partner.

For exact phrasing of options in both scenarios, please refer to supplementary materials.

### Vaccine scenario

The second part of the survey presented participants with a scenario related to vaccination options. Note that as the survey was conducted, all studied countries were beginning vaccine rollout, and vaccines were mostly available to health-care workers and certain age groups (see Fig. S1). Thus, participants were asked to imagine that there is a vaccine protecting against COVID-19 with certain characteristics available for them. We manipulated a) the information about the perceived costs for the participant (no side effects and no monetary costs *versus* flu-like side effects and costing 5% of an average local monthly income), and b) the perceived group immunization (“All/ No-one of your friends and co-workers have gotten the vaccine”). Each of the two binary variables was randomized (Table S3). We further made sure that participants were aware of the fact that vaccines also protect others by explicitly stating that this particular vaccine not only immunizes the inoculated person, but also protects their contacts. Participants were then asked to indicate how likely it is for them to get the vaccine on a slider ‘Certainly no’ to ‘Certainly yes’.

### Open ended answers

After each decision in the scenarios, participants had the option to specify their motivation for their chosen action in their own words.

### Vaccination (long-term/invasive) versus mask-wearing and testing (short-term/noninvasive) intentions in different situations

Many European countries have implemented access control to certain events, where either vaccination, a test to certify no ongoing infection (e.g., PCR tests, antigen tests), or strict mask-wearing is demanded. We asked participants to indicate whether they would comply with regulations demanding them to i) wear masks and ii) undergo a test to certify no ongoing infection, or whether they would favor being vaccinated in order to pursue the several desired activities. Participants answered on a slider ranging from absolutely favoring the test and protective gear use to absolutely favoring vaccination (coded 1–101).

### Questionnaire measures

Participants filled in i) the Anti-Mask Movement scale^13^, measuring perceived ineffectiveness and psychological reactance regarding face-masks (e.g., *Face masks are ineffective*), ii) a short Empathy scale designed to map empathetic feelings towards groups affected by COVID-19 pandemic (e.g., *I am very concerned about those most vulnerable to coronavirus*)^12^ , and iii) a scale measuring belief in COVID-19-related fake news (e.g., *The virus is a means of governments and powerful people to gain political control*). This measurement was purpose made for this study based on the most common COVID-19 related misbeliefs at the time^56^, where each of the six items represents one of the most frequent beliefs based on van Mulukom and colleagues’ systematic review of COVID-19 conspiracy-related myths^56^. The questionnaires are available here: https://osf.io/fhns8/.

All scales were translated to German, Spanish, Polish, and Czech by a native speaker and reviewed by another native speaker. The full questionnaires in all languages are available via the Open Science Framework: https://osf.io/qzscm/. Values indicating internal consistency were high for all translated scales (Table 1).

**Table 1:** Internal consistency of the Anti Mask Movement, Empathy Scale, Fake News Scale, and the two subscales of the Perceived Vulnerability to Disease Scale (PVD) as measured in this study.

Language (Sample)	Cronbach’s Alpha				
	Anti-Mask Movement	Empathy Scale	Fake News Scale	PVD: Germ aversion	PVD: Infectability
English (UK)	0.898	0.898	0.915	0.733	0.634
Czech (CR)	0.916	0.863	0.940	0.631	0.741
German (AT)	0.880	0.890	0.951	0.724	0.649
Polish (PL)	0.917	0.933	0.900	0.672	0.634
Spanish (ES)	0.887	0.927	0.925	0.583	0.639

Further, participants completed the iv) Perceived Vulnerability to Disease (PVD,^57^) Scale, which consists of two subscales: *Germ aversion* and *Infectability.* The subscale Germ aversion measures the discomfort in situations in which the possibility of a disease transmission is high (e.g., *It really bothers me when people sneeze without covering their mouth*). The subscale Infectability measures participants’ beliefs about their susceptibility to infectious diseases (e.g., *I am more likely than the people around me to catch an infectious disease*; The translations of the PVD to German, Spanish, Polish, and Czech are available on OSF from a previous project: https://osf.io/2a4rc/.

### Analysis

Mask scenario I was analyzed using binary logistic regression. Mask scenario II was analyzed using a multinomial logistic regression, where the least safe option was chosen as a reference group. The vaccine scenario was analyzed using a univariate general linear model.

In all cases, the input variables were age, sex, country of residence, and the questionnaire scores. In addition, information about the interaction partner for the mask scenario and group immunization, as well as ‘costs’ for the vaccine scenario, entered the models. The outcome variable was the scenario response. In the binary logistic regression, we employed backwards elimination (Likelihood-ratio based on maximum partial likelihood estimates) to remove non-significant predictors from the final model. For the multinomial logistic regression, variance inflation factors suggested no excessive multicollinearity (highest VIF = 1.103). The final model was selected based on AIC values (AIC_final_ = 1051.460). The final GLM for the vaccine scenario was chosen based on combined enter and backwards methods, i.e., minimizing the amount of variables while maximizing the adjusted R^2^.

To analyze preferences for vaccination mandates or other measures in different situations, univariate ANOVA was employed. We used Bonferroni adjustment throughout to correct for multiple comparisons in the post-hoc analysis. Analyses were performed in SPSS 21.0 (IBM Cop.).

The open-ended answers provided by participants to explain their choices were classified into groups based on the participant’s responses to the scenarios (*e.g.,* high vaccination intention/undecided/low vaccination intention). Two scientists independently identified recurring motives for the participants’ decisions within each of these pools and, based on abundance of and redundancies in these motives, decided on a set of existing main motives in each subgroup. They then independently coded answers as one of these motives. Discrepancies in the coding were then discussed, and motives revised and specified accordingly to avoid misunderstandings. The whole process was repeated until 100% agreement was reached. Answers were translated to English by native speakers of the respective language before coding and are available together with the quantitative data.

### Ethical statement

Participants provided informed consent prior to their anonymous participation. The project was evaluated and approved by the Ethical Committee of the Faculty of Science, Charles University, as part of GDPR regulations were followed at all times, and research was conducted in accordance with the Declaration of Helsinki and other relevant guidelines.

## Results

### Mask wearing: What predicts the first active move?

In the first mask scenario, participants first had to decide whether to take their mask off or leave it on. The results of a binary logistic regression showed a significant positive predictive value of four variables for keeping the mask on (see Table S4): i) relationship with the interacting partner (if the interaction partner is a potential co-worker, not a family member, odds ratio = 5.357), ii) higher empathy with those vulnerable to the pandemic (odds ratio = 1.083), iii) higher germ aversion (odds ratio = 1.061), and iv) higher age (odds ratio = 1.022). Additionally, we found a significant negative predictive value of v) higher disbelief in protection of masks (odds ratio = 0.925)(Table 2). Further, country of residence had a significant predictive value, where Spain, which was most affected by the pandemic at the time of data collection, was used as a reference group. Specifically, residence in Poland (odds ratio = 0.228) and the UK (odds ratio = 0.187) relatively decreased the probability of keeping the masks on. Participants sex, belief in COVID-19 related fake news, perceived own infectability, and prior information about the interaction partner had no significant predictive value. Figure S3A, B depicts percentages of participants who decided to take their mask off across countries.

**Table 2:** Summary of predictors of scenario outcomes.

Scenario	Outcome	Positive predictors	Negative predictors	Not significant predictor
Mask scenario I: Active first move	Leaving on the mask (safe, cooperative) as opposed to taking it off (putting partner at risk)	Interaction is with stranger Empathy Germ aversion Higher age	Disbelief in protection of masks Residence in PL, UK as compared to Spain	Infectability Prior information about partner Residence in other countries Sex Belief in fake news
Mask scenario II: Responding to partner taking off their mask	Keep on own mask (regardless of whether the other person is being tolerated without mask or if the other person is asked to keep using their mask)) as opposed to both partners taking off the mask (equally unsafe)	Empathy Germ Aversion Higher age	Interaction is with family member Disbelief in protection of masks Residence in CZ, PL, UK, AT as compared to Spain	Infectability Prior information about partner Sex Belief in fake news
Vaccine scenario	Intention to take the vaccine	Empathy	Financial investment and side effects Disbelief in protection of masks Belief in fake news	Immunization status of surrounding group Country Sex Germ aversion Infectability Age

The model (χ^2^ = 186.889, *p *< 0.001) predicts 79.8% of the analyzed cases correctly.

### Mask wearing part II: How to react to the partner’s move

In the second part of the mask scenario, participants had to respond to their interacting partner taking off the mask. They could choose to A) keep their mask on without protesting, B) take their mask off as well, C) protest and demand the partner to put the mask back on, or D) demand the partner to put the mask back on while taking their own mask off. Here, only a small proportion (< 5% of answers) of participants chose the option of putting only the other person at risk (D). Therefore, we omitted the category in the model to reduce data dispersion. For descriptive results on that category, see Fig. S3 C, D.

The results of a multinomial logistic regression (full model:χ^2^ = 217.648, *p* < 0.001) with the most unsafe option (both individuals taking off their masks) as a reference group revealed six variables that were significant predictors of the overall outcome behavior (Table 2): i) higher disbelief in protection of masks (χ^2^ = 69.172, *p* < 0.001), ii) whether the interaction partner is a family member (χ^2^ = 38.394, p < 0.001), iii) country of residence (χ^2^ = 36.5, *p* < 0.001), iv) germ aversion (χ^2^ = 38.394, *p* < 0.001), v) age (χ^2^ = 24.010, *p* < 0.001), and vi) empathy with those most vulnerable to the pandemic (χ^2^ = 10.626, *p* = 0.005). Participants’ sex, belief in COVID-19 related fake news, and prior information given about the interacting partner had no significant predictive value. Figure S3C, D depicts the percentage of participants choosing each of the four possible moves.

When characterizing predictors of each possible reaction in reference to the most unsafe option (B), we found that the predictors of keeping one’s mask on while tolerating the interacting partner wearing no mask (A) and of protesting the partner’s unsafe action while keeping the mask (C) (Table 2) are highly similar. Specifically, we found six significant predictors in our model when compared to the riskiest option (both participants taking off their mask):

The three variables i) higher empathy with those vulnerable to the pandemic (odds ratio for A = 1.078, odds ratio for C = 1.163), ii) higher germ aversion (odds ratio for A = 1.034; (odds ratio for C = 1.086), and iii) higher age (odds ratio for A = 1.028; odds ratio for C = 1.051) positively predicted choosing this option. Conversely, iv) higher disbelief in protection of masks (odds ratio for A = 0.929; odds ratio for C = 0.917), and v) if the interaction partner was a family member (odds ratio for A = 0.373; odds ratio for C = 0.203) negatively predicted this choice as opposed to the reference group. Further, vi) the participants’ country of residence had a predictive value; specifically, residence in Austria (odds ratio for A = 0.454; odd’s ratio for C = 0.300), Poland (odds ratio for A = 0.228; odds ratio for C = 0.213), Czech Republic (odds ratio for A = 0.253; odds ratio for C = 0.127), and United Kingdom (odds ratio for A = 0.200; odds ratio for C = 0.098) negatively predicted the choice of the mask-wearing options when compared to Spain as the most affected country.

Given that the same variables were significant predictors for both groups (note that even effect sizes are highly similar), they should be seen as general predictors of keeping one’s own mask on, independent of the partner’s previous move. Note that predictors also resemble those for keeping one’s mask proactively in scenario I (Table 2).

### Decision motives to wear or not to wear masks

Participants were given the option to explain their choices in the scenarios (see Table 2 for an overview) in their own words (*n* = 321 explanations given, AT = 64, ES = 60, CZ = 45, PL = 81, UK = 71). We broadly classified the participants who gave these answers into three groups based on the participant’s choice in the scenarios: those always wearing a mask (*n* = 221 explanations given), ‘switchers’ who mostly wear the mask with the stranger but not with the cousin (*n* = 61 explanations given), and those never wearing a mask (*n* = 39 explanations given). (Table 2).

For the group always wearing their masks, 88% and 87% of explanations given for decisions with the cousin and stranger respectively mentioned one of seven main motives: i) safety of specific individuals (cousin: 24%; stranger: 30%), ii) general safety concerns (cousin: 18%, stranger: 15%), iii) avoiding conflict, and practicality of communication (cousin: 13%, stranger: 9%), iv) personal freedom and individual choice (cousin: 9%, stranger: 6%), v) generalized responsibility during a pandemic (cousin: 8%, stranger: 7%), vi) professionalism at the workplace and legal regulations (cousin: 7%, stranger: 13%), and vii) knowing the interaction partner, or lack of prior knowledge about the interaction partner (cousin: 5%, stranger: 8%). The first and most abundant motive, safety of specific individuals (i), often included safety of the participants themselves (cousin: 93%, stranger: 89%), but also includes mentions of the partner in the current interaction (cousin: 39%, stranger: 43%), and of others outside the interaction (e.g., elderly grandparents one could carry the disease to; cousin: 25%, stranger: 31%) (subcategories non-mutually exclusive).

For those wearing the mask only in one scenario, 86% (stranger) and 77% (cousin) of all answers contained one of the following motives: i) generalized trust in family members (43%; only present in cousin condition), ii) knowing the interaction partner, or lack of prior knowledge about the interaction partner (cousin: 13%, stranger: 33%), iii) ease of social interaction (cousin: 6%, stranger: 16%), and iv) professionalism at the workplace and legal regulations (stranger: 18%, cousin: 3%). Further, some less abundant decision motives only occurred in either the stranger or cousin scenario. In the interaction with the stranger, these were: i) safety of specific individuals (6%), ii) avoidance of conflict (6%), and iii) generalized safety considerations. In the interaction with the cousin, physical discomfort with the mask was identified as the main motive in an additional 8% of the answers.

For the group never choosing to wear a mask, 96% (both cousin and stranger) of the responses were assigned one or multiple of the following three main motives: i) rationalization (e.g., that the situation will be safe or unsafe regardless of the mask; cousin: 48%, stranger: 33%), ii) ease of social interaction (both: 29%), and iii) physical discomfort of masks (cousin: 19%, stranger: 24%). Only for the interaction with the stranger, an additional 10% of explanations contained social reciprocity and joint agreement of both persons as the main decision motive.

### Predictors of vaccination intention

Following the mask scenarios, participants were presented with a hypothetical vaccination scenario (see *2.5.*). The result of the univariate general linear model (F(1, 291) = 106.568, *p* = 0.001, η_p_^2^ = 0.268) provides us with four significant predictors of vaccination intention (see Table S7, Table 2). Negative predictors were i) existing ‘costs’ of the vaccine (high financial investment and side effects *versus* neither) (F(1, 291) = 25.162, *p* < 0.001, η_p_^2^ = 0.080), ii) disbelief in mask wearing as a protective measure (F(1, 291) = 17.328, *p* < 0.001, η_p_^2^ = 0.056), and iii) belief in COVID-19 related fake news (F(1, 291) = 16.235, *p* < 0.001, η_p_^2^ = 0.053). In contrast, iv) empathy with those vulnerable to the pandemic (F(1, 291) = 9.51, *p* = 0.002, η_p_^2^ = 0.032) positively predicted vaccination intention. Immunization status of the surrounding social group (hypothetical co-workers and friends) had no effect, nor did sex, country of residence, the participants’ age, or the scales germ aversion and infectability. (Table 2).

### Decision motives of groups with high or low vaccination intention and undecided groups

A subset of participants (*n* = 171; AT = 27, ES = 26, CZ = 35, PL = 37, UK = 46) provided explanations in their own words for their decisions in the vaccine scenario. The answers were divided to three sub-groups based on the score indicating vaccination intention: pro-vaccine (scores from 61 to 101, *n* = 121 explanations given), undecided (scores from 40 to 60, *n* = 24 explanations given), and anti-vaccine (scores from 1 to 39, *n* = 26 explanations given). (Table 3).

**Table 3:** Summary of decision motives reported in open-ended answers.

Scenario	Group based on responses in scenarios	Top three decision motives (% of answers categorized as such)	Other decision motives (% of answers categorized as such)
Mask scenarios	Always wearing masks (n = 221)	Safety of specific individuals (C: 24%, S: 30%) Generalized safety concerns (C: 18%, S: 15%) Avoiding conflict and practicality of communication (C: 13%, S: 9%)	Personal freedom and individual choice (C: 9%, S: 6%) Generalized responsibility during a pandemic (C: 8%, S: 7%) Professionalism at the workplace and legal regulations (C: 7%, S: 13%) Knowing the interaction partner, or lack of prior knowledge (C: 5%, S: 8%)
	Wearing mask in only one scenario (n = 61)	Generalized trust in family members (C: 43%) Knowing the interaction partner, or lack of prior knowledge (C: 13%, S: 33%) Ease of social interaction (C: 6%, S: 16%)	Professionalism at the workplace and legal regulations (C: 3%, S: 18%)
	Never wearing mask (n = 39)	Rationalization (C: 48%, S: 33%) Ease of social interaction (C: 29%, S: 29%) Physical discomfort of masks (C: 19%, S: 24%)	
	High vaccination intention (n = 121)	Safety of specific individuals (26%; of these, 97% included protection of the participant, 68% also included protection of others) Enabling a return to ‘normality’ (18%) Trust in science and official institutions (13%),	Community-level altruistic motives (10%) Trade-off considerations about benefits and risks of vaccines (9%)
Vaccine scenario	Undecided (n = 24)	Doubts about the efficiency and safety of vaccination (40%) Safety from the disease (12%) Considerations referencing trust in science and official institutions (12%)	
	Low vaccination intention (n = 26)	Generalized distrust in vaccines (27%) Cost of the vaccine in the presented scenarios (23%) Doubts about the effectiveness of vaccines (13%)	Fear of side-effects (10%)

*C* Cousin scenario, *S* Stranger scenario.

For the pro-vaccine group, 76% of all answers contained one or multiple of five main decision motives: i) safety of specific individuals (26%; of these, 97% included protection of the participant, 68% also included protection of others), ii) enabling a return to ‘normality’ (18%), iii) trust in science and official institutions (13%), iv) community-level altruistic motives (10%), and v) trade-off considerations about benefits and risks of vaccines (9%). The remaining 22% of answers were diverse and could not be classified into a more abundant category.

For undecided individuals, 62.5% of all answers mentioned one or multiple of three main motives: i) doubts about the efficiency and safety of the vaccine (40%), ii) safety from the disease (12%), and iii) considerations referencing trust in science and official institutions (12%). The remaining answers could not be classified into categories amounting to more than 5% of all answers.

For the group with the lowest vaccination intention, 77% of the answers could be classified as one of these four motives: i) generalized distrust in vaccines (27%), ii) cost of the vaccine in the presented scenarios (23%), iii) doubts about the effectiveness of vaccines (13%), and iv) fear of side-effects (10%). The remaining answers were diverse.

### Vaccination (long-term/invasive) versus mask-wearing and testing (short-term/noninvasive) intentions in different situations

Next, we sought to describe participants’ approval for vaccination or mask-wearing and testing in order to pursue social and professional activities such as attending concerts or international travel. Here, participants could indicate on a slider if they would rather wear masks and undergo testing to certify no ongoing infection with the virus causing COVID-19 (e.g., PCR tests, antigen tests) whenever attending one of the listed events, or if they would rather get vaccinated against COVID-19 in order to pursue the activities. For all countries and situations, on average, participants leaned towards vaccination (Table S8). A one-way ANOVA of preferences for the long- or short-term solution between countries and events revealed no statistically significant difference.

## Discussion

The present study cross-nationally examined predictors of mask-wearing and vaccination intention by employing hypothetical social scenarios in the context of the COVID-19 pandemic.

Our results emphasize the importance of empathy as a positive predictor of compliance with health measures, and belief in fake-news as a negative predictor.

While the status of the interacting partner (family/co-worker) influenced decision-making, there was no effect of the prior information about them. Similarly, there was no influence of immunization status of the surrounding group on vaccination intentions. Both results contradict the ‘free-riding’ hypothesis of defying health measures^45^, even though pro-social motivation (empathy) seems to be an important factor.

### Motivations and predictors for mask-wearing decisions

When examining participants’ active first move, the majority of participants across the observed countries decided to keep the mask on (Fig. S3). For both the ‘first move’ scenario and the response to the partner’s move, predictors for deciding to keep the mask on as opposed to taking the mask off included higher empathy with groups vulnerable to the pandemic, higher age, and higher germ aversion. Conversely, beliefs in misinformation decreased the odds of choosing safer options.

When the interaction partner was presented as a family member, this markedly increased taking the mask off both as a ‘first move’ and in response to the partner’s action, whereas information about prior risky behavior of the partner had no influence on mask wearing. This points to an in-group bias instead of a rational safety consideration (as has been reported in numerous other situations, *see*^20,58^). Across scenarios, higher age positively influenced the intention to keep the mask on, corroborating results that show a small but significant age effect on health precautions^59^. While this effect is in line with heightened protection motivation due to the increased vulnerability to COVID-19 in older populations^60^, general beliefs about one’s own susceptibility to infection were not a significant predictor of mask wearing in either of our scenarios. Indeed, De Connick and colleagues (^61^, see also^62^) report that infectability perception may not be affected by age, while scores on germ aversion increased with age^61,62^. In line with this, germ aversion (a measure of disgust-triggered responses to potentially harmful germs) was a prominent predictor in every scenario, emphasizing instead the role the behavioral immune system might play in COVID-related decision making (see also^63,64^).

Interestingly, belief in COVID-19 related fake news (including high distrust towards authorities) was an important predictor for vaccination intention, but not a significant predictor of mask-wearing decisions, which is in line with previous results^65^**.** This suggests that distinct decision processes were involved in different protective behaviors, and susceptibility to fake news could affect mask-wearing and vaccination intention in two different ways. When in doubt, individuals can engage in ‘testing’ circulating information by engaging in the behaviors in question^66^. Since masks can be easily removed and put back on and the expected costs of doing either are low, individuals can engage in both options, which in turn reduces the association between the behavior and the adverse effects announced by the circulating fake news. A certain promiscuity in mask-wearing tendencies over the course of the pandemic has indeed been shown in several European countries^67^. In contrast, the psychological barrier of having an alien substance inserted in one's own body is higher, and knowing that vaccination cannot be ‘undone’ might prevent individuals from engaging in acts of ‘testing’^68,69^. In consequence, the lack of engaging in the behavior might reinforce the potency of the beliefs in circulating myths.

In both mask-wearing scenarios, we observed an influence of country of residence: We had chosen the most affected country as a reference(i.e., Spain), and indeed, residence in all other countries relatively decreased the probability to adhere to the measures. At the time of data collection, Czech Republic, the UK, and Poland were at a local minimum of case numbers (‘between waves’), whereas Austria and Spain were close to local maxima in case numbers (see^70^). This can be interpreted as partial support for the Peltzmann effect in the context of the COVID-19 pandemic, which describes risk compensation in behavior: As external risk factors become contained, risky behavior increases^71^. Overall, the observed national differences likely arose from a combination of cultural and social factors, as well as governmental restrictions and COVID-related experience in the respective areas. Notably, Spain had also enforced the most stringent mask mandates in the months prior to this study (Fig. S2).

The main set of motivations for those participants who always decided to keep their masks on were the emphasis on protection of self and others, generalized concerns, and avoiding conflict. In contrast, the most common decision motive for those wearing masks only with the potential colleague, but not with a cousin, was trust placed in family members (cf.^20^), which is particularly interesting since the prompt clearly stated that the participant had not seen the family member in a long time, and in some cases explicitly gave information on the family member’s risky behavior. This indicates that participants were more sensitive to cues of implicit relatedness than explicit risk assessment. Indeed, other (non-conscious) cues of relatedness such as phenotypic resemblance have been found to significantly affect decisions in trust games^72^.Those who never chose to wear a mask often rationalized their action by either explaining that masks are inefficient, or that the risk of infectionat an appropriate distance is nil. These rationalizations of health-threatening behaviors for both oneself and others is well-documented from smoking behavior^73^, where reasons such as “Knowing heavy smokers that lived long” or “Not everyone gets sick smoking” are commonly reported^73^. Indeed, these claims closely resemble the motives reported in this study, such as “My body has a good immune system”. Research aiming to elicit protection motivation in such cases (e.g.^74,75^) could hold interesting insights. For example, strategies to raise awareness of endangering others, using the authority of an attractive model disapproving of a given behavior, or providing people with stimuli showing that a given behavior hinders achieving life goals^75^, have yielded promising results in this area.

Second, participants never choosing to wear the mask reported the wish to facilitate the interaction by not wearing masks, for example, the fear that it could be “awkward” and the need to see faces when talking. This is of little surprise since facial expressions convey much important information in human communication^76^. Finally, comfort – a purely individual cost – was also reported as a motive to take the mask off.

### A friendly jab: Vaccination intentions and motives

Individual misinformation about the virus as well as about protective measures was a prominent negative predictor of vaccination intention, strongly emphasizing the need for tailored public science communication. As expected, higher costs of vaccines (both monetary and in terms of side-effects) decreased vaccination intention (see^77,78^). Interestingly, we found no effect of immunization status of the surrounding social group, and thus no empirical evidence for the ‘free-riding’ hypotheses (see also the discussion in reference^79^) or, conversely, for a strong influence of social conformity^52^. The only positive psychometric predictor of heightened vaccination intention was higher empathy with those most vulnerable to the pandemic.

Overall, and across all observed countries, a larger fraction of our sample preferred to get a vaccine protecting against COVID-19 rather than undergo testing and wearing face-masks in order to attend social and professional events, and medical appointments (Table S8). Nevertheless, those reporting low vaccination intentions in the scenarios provided interesting insights. Both undecided and anti-vaccine groups reported doubts regarding the effectiveness of the vaccine and fears of side-effects (e.g., *“experimental drugs”; “would need to know other things about this vaccine trials and long term side effect*”)*.* Often, the medical meaning of “long-term side effects” seemed to be misunderstood.. These recurring motives in our sample are an alarming signal regarding public science communication and misinformation, and call for an increased effort in science education on all societal levels^80^.

Conversely, participants with a high vaccination intention reported their own protection, but also prominently the protection of others (e.g., their elderly grandparents) as main motives. A smaller proportion also referenced their trust in science and institutions, which directly relates to the often referenced trust in public institutions as a compliancy-promoting factor^42^. Perhaps this can be seen as a counterpart to the effect of COVID-19 related fake news on vaccination intention, since these often include theories that powerful institutions and governmental actors may be involved in conspiracies (see also the items from our scale). While personal protection has been featured in many campaigns and has been shown to be an important motivation both in this study and in previous works^81^, our results additionally urge for a more prominent inclusion of pro-social motives in public appeals (see also^82,83^).

### Limitations

Online data collection enabled us to reach a sufficiently large and comparable cross-national sample during a pandemic. However, this approach also has its limitations. While our study differs from previous questionnaire-based work by asking participants to vividly imagine social scenarios including their acts, their decisions remained hypothetical, and the scenarios lacked much of the complexity and nuances of real-life social interactions and group dynamics. As such, our results may not be representative of the multitude of social situations and decisions citizens encounter in their everyday life. Rather, they enable us to roughly estimate reactions to health-related measures based on individual characteristics and social circumstances, and the insights may be useful in designing public health interventions tailored to appeal to self-reported motives. Furthermore, these data provide a starting point to generate hypotheses and design studies on smaller samples in real-life settings. For example, our mask scenarios could be adopted in analogous studies, where an accomplice takes the role of the fictive partner. Importantly, intended behaviors (as typically reported in online studies) and actual behavioral outcomes can differ. Incorporating variables such as political influences, governmental incentives, need for official documents, and an account of public health debates in a given area could help in closing the gap between intended and actual behavioral responses^84–89^.

The current study benefits from its cross-national setting, increasing the generalizability of the findings. However, the individual sub-samples are not large enough to be representative for each given country, particularly given a larger proportion of female respondents and a bias towards higher education. Unfortunately, the sampling method, and exclusion of participants (failing attention checks) itself limits the sample to persons with internet access and sufficient experience with formalized questions.

Moreover, our findings should be interpreted in the temporal context of the COVID-19 pandemic. At the time of data collection, vaccines were thought to end the pandemic by protecting individuals from catching and transmitting the virus, and eventually achieving herd immunity in the population^90^. However, available vaccines were limited and had only started to be distributed to certain groups with high vulnerability. Since then, vaccines have become widely available in the studied countries, but while the vaccines’ protection against severe disease is still evident^91^, herd immunity is not communicated as the desired outcome anymore since it may be impossible to reach^92,93^. Progressing vaccination rates have in turn affected public mask-wearing behaviors^94^, and alertness and general interest has shifted^95^.

## Conclusion

The present study identifies predictors and motives for safety and ‘common good’ considerations in social interactions in the context of the COVID-19 pandemic.

Whereas the decision to wear a mask was influenced by family status of the interacting partner (less likely with cousin than co-worker), prior information about their trustworthiness and safety status did not prove influential. This emphasizes the importance of in-group feelings as opposed to rational moves predicted by game theory. Furthermore, pro-social psychological characteristics and motivations, as well as germ aversion, were dominant predictors for both mask-wearing and vaccination intentions. Therefore, appealing to the protection of others, appealing to a feeling of disgust regarding germs and the disease, and enhancing identification with local communities could be an effective prevention against health-threatening behaviors.

Taken together, our analysis of predictors and motives of responses to hypothetical scenarios provides a starting point towards evidence-based campaigns and in-person studies of social behavior in potentially infectious situations.

## Supplementary Information

Supplementary Information.

## Data Availability

The data of this study are openly available at https://osf.io/qzscm.
