# Supplementary material for: Predictors and motives for mask-wearing behavior and vaccination intention
Source: Sci Rep. 2023 Jun 25;13:10293. doi: 10.1038/s41598-023-37072-6 (PMC10290991; doi:10.1038/s41598-023-37072-6)
Supplement: Supplementary file 1 — Supplementary Information. [file 41598_2023_37072_MOESM1_ESM.docx]

**Supplementary Material**

**Content**

**1. Supplement to Methods (**[p2](#supl_to_meth)**)**

Table S1. Sociodemographic information ([p2](#TabS1))

Figure S1. Vaccine availability over time([p3](#news1))

Figure S2. Mask mandates over time ([p4](#news2))

Instruction Mask scenario ([p5](#newfirstintro))

Table S2. Information conditions about interacting partner ([p5](#TabS2))

Mask scenario 1 ([p6](#MaskSce1Answ))

Mask scenario 2 ([p6](#MaskSce2Answ))

Vaccine scenario/ Table S3: Possible vaccine scenario prompts ([p8](#VaccSce))

Links to all items and questionnaires ([p9](#FullListItems))

**2. Supplement to Results (**[p10](#SuplRes2)**)**

Figure S3. Percentages of participants’ chosen reactions in the mask scenarios ([p10](#FigS1))

Model outcomes: Supplementary tables ([p11](#TabS4))

*Table S4. Model outcomes of mask scenario 1* ([p11](#TabS4))

*Table S5. Predictors of keeping one’s own mask while tolerating the partner without mask* ([p12](#TabS5))

*Table S6. Predictors of insisting that the partner puts their mask back on* ([p13](#TabS6))

*Table S7. Model outcomes of the vaccine scenario* ([p14](#TabS7))

*Table S8. Vaccination versus mask-wearing and testing intentions* (p15)

**1.** **Supplement to Methods**

**Table S1. Sociodemographic information**

|  |  | Overall | Czech Republic | Poland | United Kingdom | Spain | Austria |
| --- | --- | --- | --- | --- | --- | --- | --- |
| Sex | Female | 183 | 33 | 48 | 37 | 31 | 34 |
|  | Male | 113 | 23 | 29 | 24 | 21 | 16 |
| Age (mean ± SD) | | 35.27  ± 13.28 | 35.48  ± 13.41 | 34.97  ± 13.56 | 33.3  0±12.54 | 34.85  ± 14.24 | 38.36  ± 12.49 |
| Education (%) | Compulsory school | 2.7 | 9.1 | 1.3 | 0 | 0 | 4.0 |
|  | Secondary school | 24.4 | 36.4 | 23.4 | 26.2 | 11.5 | 24.0 |
|  | Finished apprenticeship | 6.1 | 1.8 | 5.2 | 3.3 | 19.2 | 2.0 |
|  | University degree – BA/BSc | 30.8 | 18.2 | 36.4 | 49.2 | 26.9 | 18.0 |
|  | University degree – MA/MSc/Mag | 31.5 | 29.1 | 33.8 | 19.7 | 38.5 | 38.0 |
|  | University degree – PhD/Dr. | 4.4 | 5.5 | 0 | 1.6 | 3.8 | 14.0 |

**
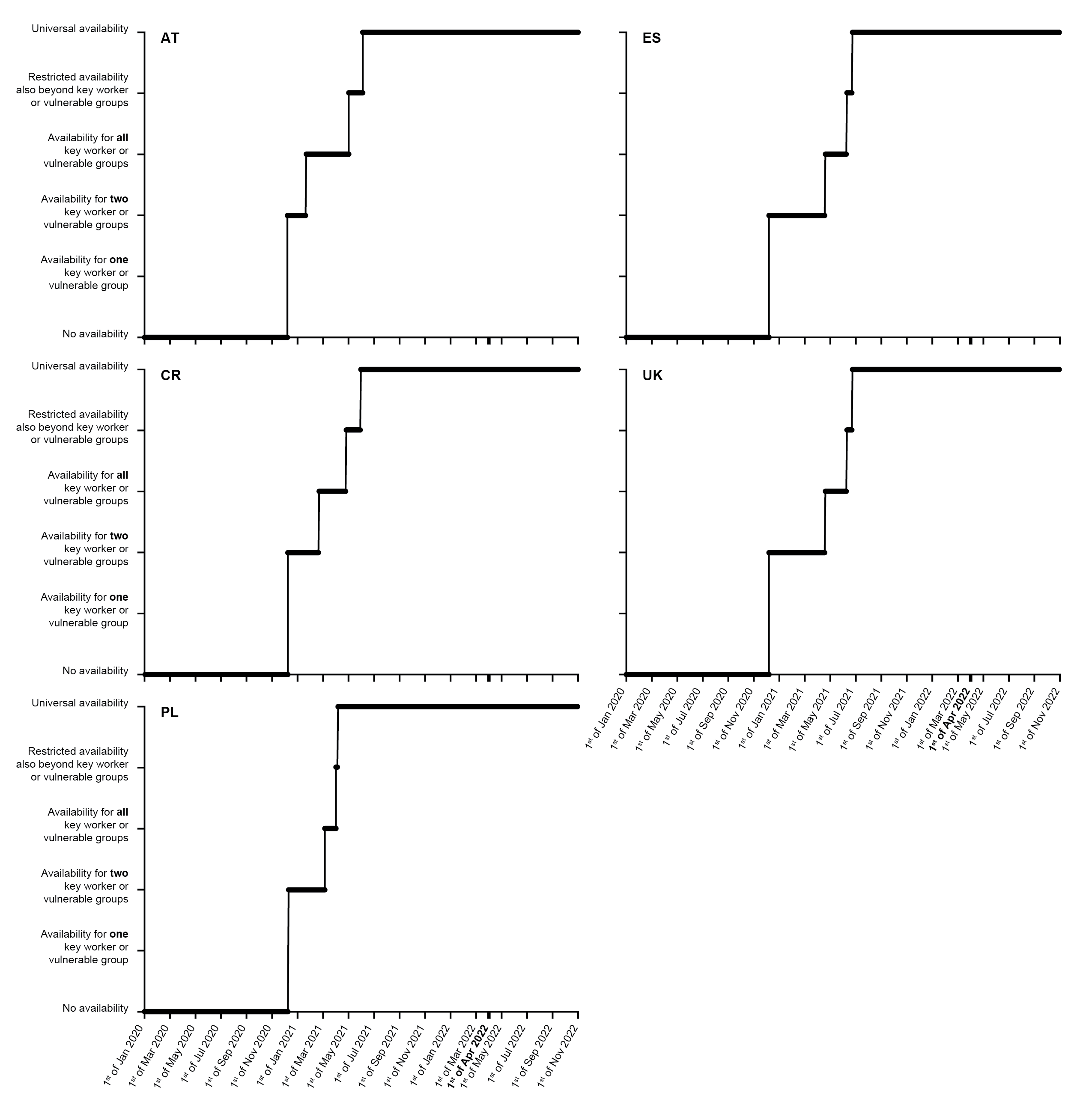
Figure S1. Vaccine availability over time.** At the time of data collection (April 2022), vaccines were only available to selected groups in all studied countries. Source: Oxford COVID-19 Government Response Tracker (<https://github.com/OxCGRT>), Hale et al., 2022.

**Figure S2. Mask mandates over time.** Mask mandates in the studied countries were present but moderate at the time of data collection. However, in the months prior to the study, stringency of mandates varied between the studied countries and over time. Source: Oxford COVID-19 Government Response Tracker (<https://github.com/OxCGRT>), Hale et al., 2022.

**
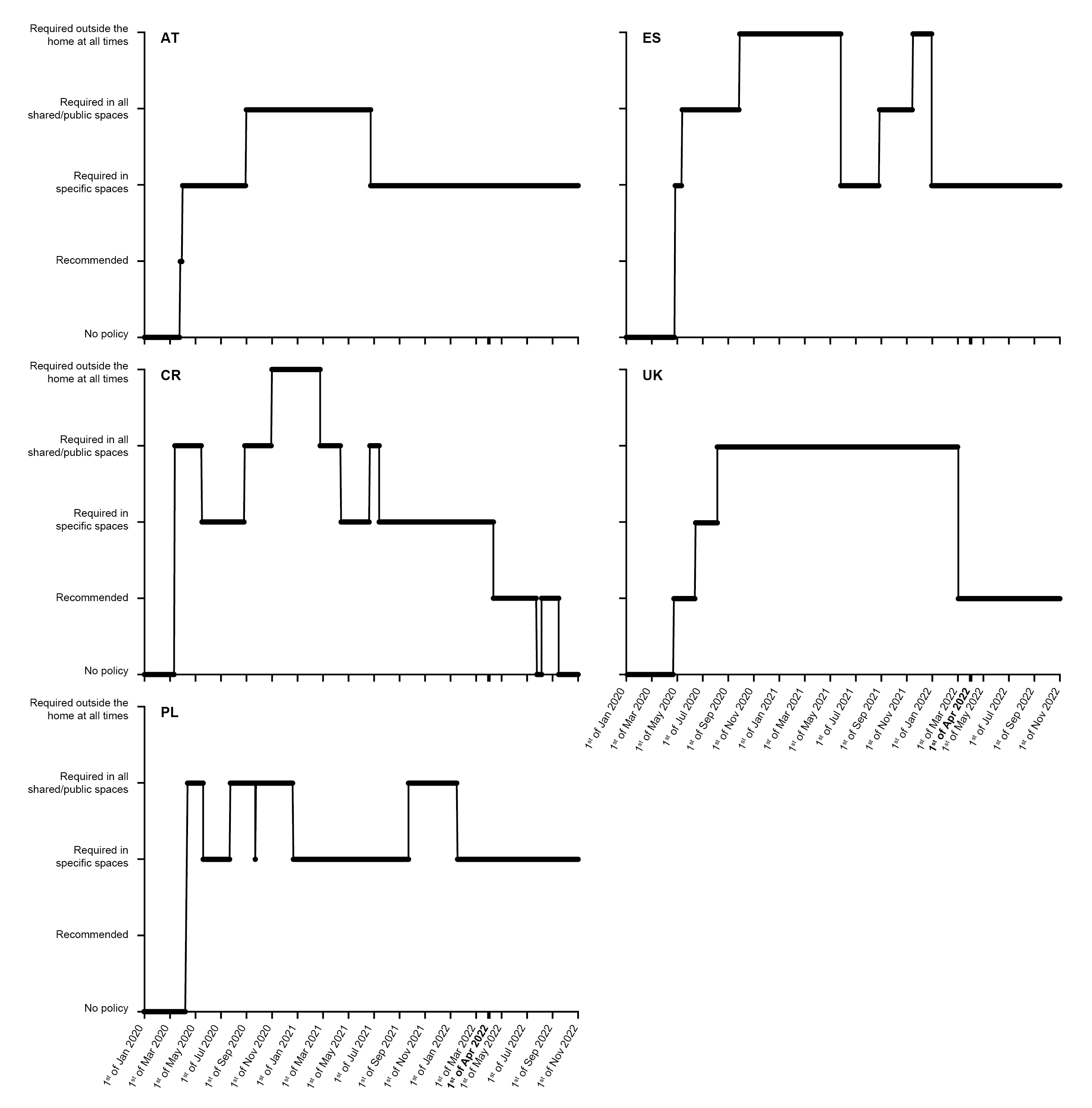
**

**First instruction Mask scenario**

Participants were given scenarios where a) status of the interacting partner (family member/stranger), and b) prior information about the interacting partner were manipulated. They first received the following prompt: “Please imagine that you are asked to meet a cousin/potential coworker”, followed by one of three prompts about the interacting partner (Table S2).

Participants were then asked: “You will meet for about an hour indoors. How would you decide in the following situations?”

**Table S2. Information conditions about interacting partner in the mask scenario, where the variables (i) status of partner (family/stranger) and (ii) information about partner were manipulated**

|  | Please imagine that you are asked to meet a: | |
| --- | --- | --- |
| Information about the interaction partner | **Cousin** | **Potential coworker** |
| *No information* | You have not heard anything about him/her for years. | You don’t know anything about this person yet. |
| *Trustworthy, implicating low infection risk* | You have been told by another relative that they are an honest and organized person. | You have been told by your colleague that they are an honest and organized person. |
| *High infection risk* | You have been told stories by another relative of their disorganized and out-going personality. | You have been told stories by your colleagues of their disorganized and out-going personality. |

**Prompt and answering options** **Mask scenario 1:**

On the following page, participants received the following prompt and answering options:

*After you enter the room, you take your jacket off and seat yourself by a small coffee table. Do you:*

*A) Keep the face-mask properly fixed on your face to ensure safety and compliance with the regulations.*

*B) Decide to take the face-mask off to ensure comfort and ease of interaction with [your cousin/the other person].*

In this scenario, option A) represents both the safe and cooperative option, whereas B) is a unilateral decision that puts the interacting partner at risk.

**Prompt and answering options** **Mask scenario 2:**

After participants answered the previous question, they were confronted with the following scenario:

*Imagine that, in the same scenario, the other person decides to take the mask off their face to allow for comfort and ease of interaction with you. Do you:*

1. *Keep your mask on regardless of their action and proceed to discuss the matter of your meeting even though you know you are put at risk of infection.*
2. *Decide to take your mask off, too, so you ensure comfort and ease of interaction with [the other person/ your cousin].*
3. *You insist that [the other person/ your cousin] will put the mask back on to ensure your own safety, and threaten to leave the meeting if they fail to comply.*
4. *You suggest that [the other person/ your cousin] puts their mask back on, but you decide to take your mask off to ensure comfort and ease of interaction with [the other person/your cousin].*

In this scenario, in option A) participants gain no comfort themselves, but are put at risk of infection, which is the opposite of option D), where the participant takes of the mask but insists for the other person to wear it: Here, the participant is at low risk of infection but gains the comfort of not wearing a mask. In option B), both partners are equally put at risk of infection, and in option C), both partners would be safe. As opposed to the first mask scenario, here, the safest option requires active action of the participant against the fictional interacting partner.

**Vaccine scenario**

**Table S3. Possible vaccine scenario prompts resulting from randomizing the two binary variables costs and group immunization.**

| *Vaccine scenario prompts* | **High costs** | **Low costs** |
| --- | --- | --- |
| **High group immunization** | Imagine there is a vaccine on the market, which leads to flu-like side effects for a week and costs *[5 % of an average monthly income in local currency]*. If you get the vaccine, you are not only effectively immune against the virus yourself, but you also cannot transmit it to others anymore. All your friends and co-workers have gotten the vaccine already. | Imagine there is a vaccine on the market, which has no side effects and is free. If you get the vaccine, you are not only effectively immune against the virus yourself, but you also cannot transmit it to others anymore. All your friends and co-workers have gotten the vaccine already. |
| **Low group immunization** | Imagine there is a vaccine on the market, which leads to flu-like side effects for a week and costs *[5 % of an average monthly income in local currency]*. If you get the vaccine, you are not only effectively immune against the virus yourself, but you also cannot transmit it to others anymore. No-one from your co-workers and friends has yet been vaccinated. | Imagine there is a vaccine on the market, which has no side effects and is free. If you get the vaccine, you are not only effectively immune against the virus yourself, but you also cannot transmit it to others anymore. No-one from your co-workers and friends has yet been vaccinated. |

**Full list of all items answered: See** <https://osf.io/qzscm/>

**Purpose-made COVID-19 related Belief in Fake News questionnaire:** <https://osf.io/fhns8/>

**Purpose-made translations to German, Czech, Polish, and Spanish of the Empathy scale by Pfattheicher et al. (2021):** <https://osf.io/wp9k5/>

**Purpose-made translations to German, Czech, Polish, and Spanish of the ‘anti- mask’ scale (perceived ineffectiveness & psychological reactance) by Taylor & Asmundson (2021):** <https://osf.io/6ra4y/>

**2.** **Supplement to Results**

**Figure S3.** A,B: Percentages of participants deciding to take the mask off (dark) versus keeping the mask on (light) across countries in (A) the condition where participants meet a family member and (B) the condition where they meet a stranger. C, D: Percentages of participants’ chosen reactions after the interacting partner actively took off their mask for (C) the interaction with the cousin and (D) with a stranger.


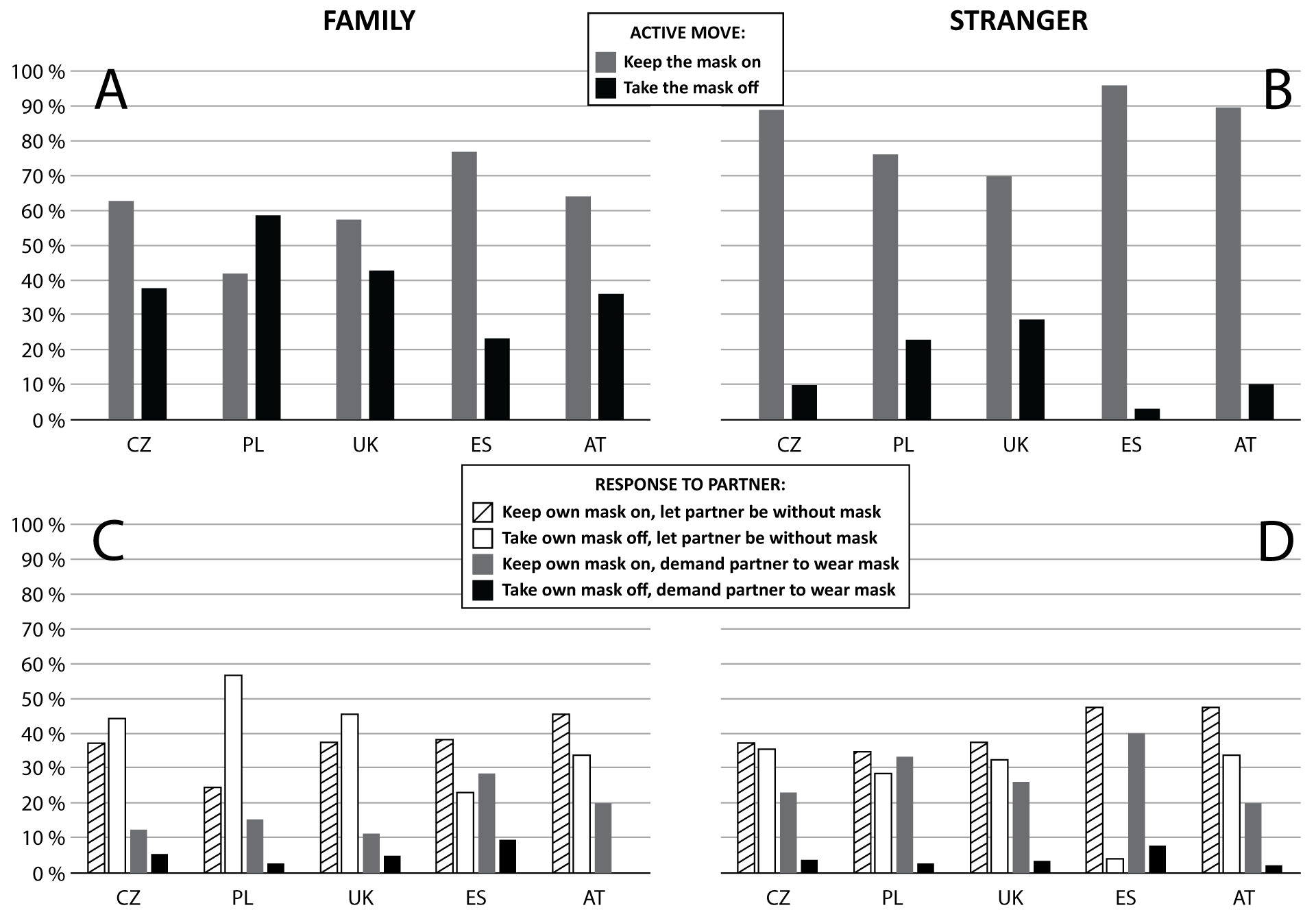


**Model outcomes: Supplementary tables**

**Table S4. Model outcomes of mask scenario 1 (first move)**

| Variable | B | S.E. | p | OR | 95% CI |
| --- | --- | --- | --- | --- | --- |
| Partner = Stranger | 1.678 | 0.226 | <0.001 | 5.357 | 3.373-8.507 |
| Empathy | 0.079 | 0.036 | 0.025 | 1.083 | 1.01-1.161 |
| Germ Aversion | 0.059 | 0.014 | <0.001 | 1.061 | 1.033-1.089 |
| Age | 0.022 | 0.009 | 0.019 | 1.022 | 1.004-1.04 |
| Anti-Mask | -0.078 | 0.010 | <0.001 | 0.925 | 0.906-0.944 |
| Countries |  |  |  |  |  |
| Czech Republic | -0.659 | 0.411 | 0.109 | 0.517 | 0.231-1.158 |
| Austria | -0.780 | 0.430 | 0.070 | 0.459 | 0.197-1.065 |
| Poland | -1.476 | 0.380 | <0.001 | 0.228 | 0.108-0.482 |
| UK | -1.675 | 0.398 | <0.001 | 0.187 | 0.086-0.409 |
| Constant | 0.327 | 0.734 | 0.657 | 1.386 |  |
| *The stranger was used as reference interaction partner, Spain as the reference country.*  *The information is provided for membership in the group that keeps the masks on*  *OR – odds ratio* | | | | | |
|  | | | | | |

**Selection of the final model:**

**Table S5. Model outcomes of mask scenario 2: Predictors of keeping one’s own mask while tolerating the partner without mask**

| Variable | | B | S.E. | p | OR | 95% CI |
| --- | --- | --- | --- | --- | --- | --- |
| Intercept | | 1.416 | 0.764 | 0.064 |  |  |
| Empathy | | 0.075 | 0.037 | 0.045 | 1.078 | 1.002-1.16 |
| Germ Aversion | | 0.034 | 0.014 | 0.013 | 1.034 | 1.007-1.062 |
| Age | | 0.028 | 0.010 | 0.004 | 1.028 | 1.009-1.048 |
| Anti-Mask | | -0.074 | 0.011 | <0.001 | 0.929 | 0.91-0.949 |
| Partner = Family member | | -0.987 | 0.227 | <0.001 | 0.373 | 0.239-0.582 |
| Country | Austria | -0.789 | 0.437 | 0.071 | 0.454 | 0.193-1.071 |
|  | Czech Republic | -1.376 | 0.411 | 0.001 | 0.253 | 0.113-0.565 |
|  | Poland | -1.478 | 0.396 | <0.001 | 0.228 | 0.105-0.495 |
|  | United Kingdom | -1.610 | 0.412 | <0.001 | 0.200 | 0.089-0.448 |
| *The reference category is the least safe option where both parties take their masks off*  *Spain was used as the reference country.*  *OR – odds ratio* | | | | | | |

**Table S6. Model outcomes of mask scenario 2: Predictors of insisting that the partner puts their mask back on**

| Variable | | B | S.E. | p | OR | 95% CI |
| --- | --- | --- | --- | --- | --- | --- |
| Intercept | | -1.417 | 0.949 | 0.135 |  |  |
| Empathy | | 0.151 | 0.048 | 0.002 | 1.163 | 1.058-1.279 |
| Germ aversion | | 0.082 | 0.017 | <0.001 | 1.086 | 1.05-1.123 |
| Age | | 0.050 | 0.011 | <0.001 | 1.051 | 1.03-1.074 |
| Anti-Mask | | -0.087 | 0.013 | <0.001 | 0.917 | 0.894-0.94 |
| Partner = Family member | | -1.593 | 0.273 | <0.001 | 0.203 | 0.119-0.347 |
| Country | Austria | -1.203 | 0.488 | 0.014 | 0.300 | 0.115-0.782 |
|  | Poland | -1.546 | 0.440 | <0.001 | 0.213 | 0.09-0.504 |
|  | Czech Republic | -2.062 | 0.480 | <0.001 | 0.127 | 0.05-0.326 |
|  | United Kingdom | -2.326 | 0.475 | <0.001 | 0.098 | 0.038-0.248 |
| *The reference category is the least safe option where both parties take their masks off*  *Spain was used as the reference country.*  *OR – odds ratio* | | | | | | |

| Variable  **Table S7. Model outcomes of the vaccine scenario** | M (SD) | df | Mean Square | F | p | Partial Eta Squared |
| --- | --- | --- | --- | --- | --- | --- |
| Corrected Model |  | 4 | 20819.756 | 26.766 | <0.001 | 0.269 |
| Intercept |  | 1 | 82815.059 | 106.468 | <0.001 | 0.268 |
| Vaccine cost |  | 1 | 19572.153 | 25.162 | <0.001 | 0.080 |
| Anti-Mask | 30.73 (11.37) | 1 | 13478.433 | 17.328 | <0.001 | 0.056 |
| Fake News | 12.40 (6.749) | 1 | 12628.411 | 16.235 | <0.001 | 0.053 |
| Empathy | 12.38 (3.029) | 1 | 7397.207 | 9.510 | <0.001 | 0.032 |
| Error |  | 291 | 777.839 |  |  |  |
| ^a^ *Computed using alpha=.05*  *R^2^ = .504; adjusted R^2^= .259* | | | | | | |

**Table S8. Vaccination (long-term/invasive) versus mask-wearing and testing (short-term/noninvasive) intentions in different situations.**

Mean ± SD indicating the preference to wear masks and get tested to attend an event (lower values) or to get vaccinated (higher values) on a slider (1 to 101). The values in parenthesis indicate the proportion of individuals who scored above 51 (lean towards vaccination).

| Country | Appointment with a medical practitioner | Work-place with possibility to spread the disease | Traveling abroad | Eating out (restaurant) | Social event (*e.g.* concert) |  |
| --- | --- | --- | --- | --- | --- | --- |
| CZ | 61±43 (57%) | 76±39 (73%) | 78±35 (73%) | 70±41 (70%) | 69±42 (66%) |  |
| PL | | 72±38 (74%) | 75±35 (81%) | 76±36 (79%) | 78±36 (79%) | 73±39 (75%) |
| UK | | 72±40 (71%) | 77±36 (77%) | 78±39 (75%) | 74±39 (74%) | 80±33 (82%) |
| ES | | 75±37 (69%) | 86±30 (87%) | 88±28 (89%) | 82±32 (73%) | 84±33 (78%) |
| AT | 76±38 (74%) | 85±31 (84%) | 86±31 (82%) | 82±35 (78%) | 85±32 (86%) |  |
